# Systemic Level of Oxidative Stress during Orthodontic Treatment with Fixed Appliances

**DOI:** 10.1155/2019/5063565

**Published:** 2019-05-23

**Authors:** Vito Kovac, Borut Poljsak, Giuseppe Perinetti, Jasmina Primozic

**Affiliations:** ^1^Faculty of Health Sciences, University of Ljubljana, Slovenia; ^2^Private Practice, Nocciano (PE), Italy; ^3^Medical Faculty, University of Ljubljana, Slovenia

## Abstract

The aim of the study was to assess the level of selected systemic oxidative stress parameters during the first week of orthodontic treatment with fixed appliances. Fifty-four males with malocclusion and having a similar lifestyle were randomized using a computer based procedure and allocated to either the treatment group (TG; n=27;24.6 ± 1.7 years) or control group (CG; n=27;24.7 ± 1.7 years). Capillary blood was collected at baseline and 6 hours, 24 hours, and 7 days after archwire insertion. At the same time points, capillary blood was retrieved in the CG. In order to determine the oxidative stress, both the reactive oxygen species (ROS) formation and the antioxidative defense (AD) potential were measured using the ROS testing and oxygen free radicals defense (equivalent to antioxidant defense) testing, respectively, by a blinded operator. The ratio between ROS and AD (ROS/AD) was calculated and data were analyzed using nonparametric tests. No drop-outs or harms were detected. At baseline, neither ROS (1.54 [1.22; 2.12] and 1.74 [1.40; 2.01] for the TG and CG, respectively), AD (1.19 [0.66; 1.50] and 1.19 [0.57; 1.42] for the TG and CG, respectively), nor ROS/AD levels were significantly different (p>0.05). After 24 hours, the ROS level significantly increased in the TG (2.05 [1.71; 2.26]) and was higher compared to the CG ROS level (1.67 [1.29; 1.95]; p=0.025), while for the AD level, no marked between and within group differences were detected. A notable change of ROS/AD ratio was observed over time only within the TG (p=0.026). Moreover, a significantly higher ROS/AD ratio was detected 24 hours after archwire insertion in the TG compared to the CG (2.69 [1.44; 3.89] and 1.79 [1.45; 2.35], respectively), followed by a decrease. Orthodontic treatment with fixed appliances might induce systemic oxidative stress in the short-term, since ROS levels and ROS/AD levels are normalized within 7 days after archwire insertion.

## 1. Introduction

The oral cavity is subjected to various external factors, including dental materials that have substantial oxidizing potential and have the ability to generate reactive oxygen species (ROS) [1]. Increased reactive oxygen species (ROS) cause oxidative stress, which is defined as the imbalance between ROS and antioxidant defense (AD) in favor of the former. During orthodontic treatment with fixed appliances, the subjects are exposed to heavy metals released from corroded appliances, which might increase the levels of ROS through metal-catalyzed free radical reactions (Fenton and Fenton-like reactions). Many metal ions such as chromium undergo redox cycling, thus directly producing ROS [2]:

(1) $$\begin{array}{c} \left(\;{Mn}^{+}\;\right)+H_{2}O_{2}\to \left(\;M^{n+1}\;\right)+{OH}^{-}+{OH}^{•} \end{array}$$

Moreover, during orthodontic treatment various inflammatory mediators (i.e., cytokines) causing aseptic inflammation in the periodontal ligament are being released after mechanical force application to the teeth inducing a cascade of reactions in the periodontal tissue, which leads to tissue remodeling and tooth movement. Since there is sound evidence indicating that periodontal inflammation is one of the main sources of ROS in the mouth [3], it is plausible that also aseptic inflammation might be associated with oxidative stress induced damage.

Several in vitro studies showed that both orthodontic brackets [4] and archwires [2] induce oxidative stress, associated with heavy metals release. In vivo studies that aimed to assess either salivary biomarkers [5, 6] of oxidative stress or biomarkers in the gingival crevicular fluid [7], reported different results. On the one hand Olteanu et al. [6] and Buczko et al. [5] reported that orthodontic treatment modifies the oxidative-antioxidative balance in the patients' saliva. In particular, Olteanu et al. [6] demonstrated that markers of oxidative stress (ceruloplasmin and malondialdehyde) increased to their highest levels 24 hours after orthodontic appliance insertion and decreased back to their initial levels 7 days after insertion. Similarly, Buczko et al. [5] evidenced a marked increase in salivary oxidative stress biomarkers one week after orthodontic appliance insertion and a decrease to normal values at the 24-week follow-up. On the other hand Atung Ozcan et al. [7] concluded that the levels of examined oxidative stress biomarkers did not change after one and six months of orthodontic treatment.

The varying results might be due to the different methodologies used and due to the different materials of orthodontic appliances to which the subjects were exposed. Moreover, the use of single biomarkers for estimating the oxidative stress is limiting, since oxidative stress is a result of an imbalance between ROS and AD in favor of the former [8, 9]. Therefore, the ratio between ROS and AD appears to be a more accurate indicator of oxidative stress [10]. To establish the complex relationship between ROS and AD direct and indirect methods can be used [11]. Direct methods relate to ROS measurements of superoxide, H2O2, OH^•^. These species are very reactive and their quantitation can be assessed only with electron paramagnetic resonance. Therefore, indirect methods are usually used, which include measurement of the balance between ROS and AD and measurements of each antioxidant separately (i.e., catalase, superoxide dismutase, vitamin C, reduced glutathione, vitamin E, etc.). The main limitation of the latter is that it does not assess the synergistic effect between different antioxidants [11].

Apart from the above-mentioned in vitro and in vivo studies of oxidative stress biomarkers changes in the local environment due to exposure to orthodontic fixed appliances, there is still paucity of data regarding oxidative stress induction at the systemic level during orthodontic treatment. Therefore, the aim of the present study was to assess the systemic level of oxidative stress during orthodontic treatment with fixed appliances, determined from capillary blood samples. The hypothesis tested was that selected oxidative stress parameters in capillary blood do change during the first week of orthodontic treatment with fixed appliances.

## 2. Material and Methods

### 2.1. Subjects and Study Design

Ethical approval for this study was gained (No. 0120-523/2018/8) from the National Medical Ethics Committee and informed consent was obtained from all subjects before inclusion. The study protocol was designed and performed following the Declaration of Helsinki for medical research involving human subjects. The data used to support the findings of this study are available from the corresponding author upon request.

A group of 54 male subjects aged between 19.7 and 28.2 years who were seeking orthodontic treatment at the Department of Orthodontics of the University Medical Centre of Ljubljana, Slovenia, due to mild crowding and teeth malalignment were recruited based on a preliminary questionnaire regarding their lifestyle habits. Subjects with oral pathology (including periodontal disease), poor oral hygiene, and known allergies as well as smoking subjects or subjects undergoing any pharmaceutical therapy, including food additives with antioxidant properties intake, were excluded. Females were not included due to possible false results as a consequence of hormonal fluctuation. Randomization was performed according to a computer based procedure having groups of equal numerosity. Twenty-seven subjects were allocated to the treatment group (TG, aged 24.6 ± 1.7 years), while the control group (CG, aged 24.7 ± 1.7 years) consisted of 27 age-matched subjects. No subject left the study.

During the study, the subjects of both groups were asked to follow a similar diet regimen (3 portions [400 g] of fruit and vegetable/day, avoidance of antioxidant supplements, and no alcohol intake) and to perform very similar activities (avoidance of extreme sport activities and sun exposure; avoidance of nocturnal life).

The fixed orthodontic appliance used in the TG was composed by stainless steel brackets (Gemini brackets, 3M Unitek; USA) attached to the upper and lower teeth and two Nickel-Titanium archwires (3M Unitek; USA) inserted in the bracket's slots.

For the evaluation of oxidative stress the balance between ROS and AD was assessed from capillary blood. The FORT (free oxygen radicals testing) and FORD (free oxygen radicals defense) assays were performed as previously described [12], using a dedicated spectrophotometer Free Oxygen Radical Monitor (FORM®, CR 3000, Callegari, Parma, Italy). Blood samples of 50 *μ*l for FORD and 20 *μ*l for FORT were collected in a sterile regimen from the tip of the subject's finger into a heparinized tube, mixed with provided reagents, centrifuged, and analyzed in the spectrophotometer by measuring light absorption at a wavelength of 505 nm. FORT and FORD values were measured immediately after blood collection. The FORT test results are given as FORT units (0,26 mg/l H2O2), while the results of the FORD test are expressed as mmol/l of Trolox (6-hydroxy-2,5,7,8-tetramethylchroman-2-carboxylic acid; a water-soluble analog of vitamin E). Principles of the determination of oxidative stress in human blood using FORD and FORT tests were previously described [13–15]. FORT and FORD analyses were performed by a blinded operator.

Capillary blood was collected before the insertion of the fixed orthodontic appliance and at 6 hours, 24 hours, and 7 days' time point. At the same time points, blood was collected and analyzed also from the matched controls. To exclude any possible influence of periodontal inflammation on the measurements of oxidative stress parameters, two weeks before the beginning of the study, all the participants were instructed regarding oral hygiene activities. At baseline, the periodontal status was assessed by measuring probing depth at six sites around every erupted tooth of each subject. Furthermore, the bleeding on probing index was used at each time point to determine the presence of inflammation.

### 2.2. Sample Size Calculation

Sample size of at least 26 subjects for each group was needed to detect an effect size coefficient of 0.8 (which is regarded as “large effect” [16]) for the measured parameters in any comparison between the groups, with an alpha set at 0.05 and a power of 0.80.

### 2.3. Statistical Analysis

The Statistical Package for Social Sciences Software release 20.0 (SPSS Inc., Chicago, Illinois, USA) was used for data analysis. The balancing of experimental groups by age was tested with a Mann-Whitney U-test. After testing the normality of the data with the Shapiro-Wilk test and Q-Q normality plots and the equality of variance among the datasets using a Levene test, nonparametric methods were used for data analysis.

A Friedman test was used to assess the significance of the differences in every parameter (FORT, FORD, and FORT/FORD ratio) over the time points within each group. When significant interactions were seen, a Bonferroni-corrected Wilcoxon test was used for pairwise comparisons. A Mann-Whitney U-test was used to assess the significance of the differences in every parameter between the two groups within each time point.

The results were considered to be significant at p-values below 0.05.

The intra-assay and inter-assay coefficients of variation were reported to be 3.7% and 6.2%, respectively, for the FORT and 4.2% and 6.6%, respectively, for the FORD [12].

## 3. Results

The results of the FORT and FORD assays for the TG and CG group at different time points are reported in Table 1. At baseline, no significant differences were detected between the TG and CG, neither for FORT (p>0.05) nor FORD (p>0.05) levels.

The FORT level in the TG increased to significantly higher values than those in the CG (p=0.025) at the 24 hours' time point, and decreased to normal values similar to those seen in the CG at the 7 days' time point. Although a decrease of the FORD level was detected in the TG at the 24 hours' time point, this was not statistically significantly different from the CG.

A significant change of the FORT level over time was seen within the TG (p=0.026), while no notable changes were detected for the FORD level (p>0.05). In the CG, neither FORT nor FORD levels changed markedly over time (P>0.05).

**Figure 1:**
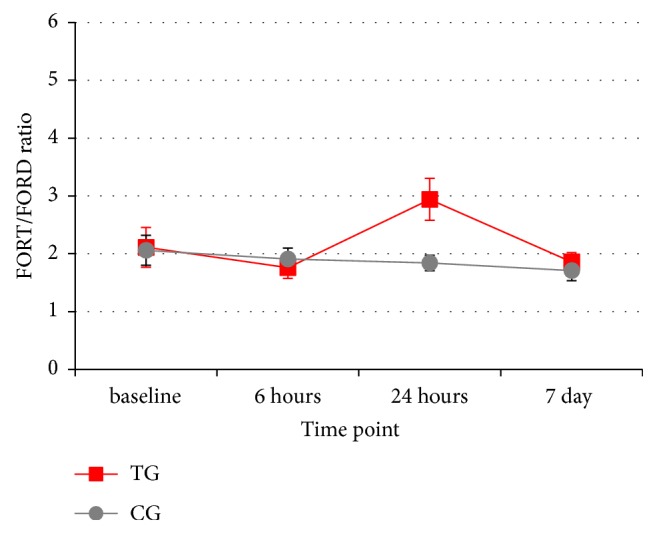
Mean values and standard errors of the longitudinal changes of the FORT/FORD ratio in the treated (TG) and control (CG) groups.

The FORT/FORD ratio, expressing the balance between ROS and AD is represented in Figure 1. At baseline and 6 hours, no significant differences regarding the FORT/FORD ratio were observed between the TG and CG (p=0.897 and p=0.528, respectively). At 24 hours, the FORT/FORD ratio increased significantly in the TG as compared to the CG (p=0.044). Finally, at the 7 days' time point, no significant differences regarding the FORT/FORD ratio were measured between the two groups (p=0.299). None of the subjects had signs of periodontal disease/inflammation over the observational period.

## 4. Discussion

It has been postulated, that orthodontic treatment with fixed appliances might play an important role in inducing oxidative stress and related damage [1]. Until recently, only local environment levels of ROS and/or antioxidant defense were assessed during orthodontic treatment, by examining either saliva [5–7, 17] or the gingival crevicular fluid [7]. To our best knowledge, the present study is the first attempt to determine the oxidative stress induced at the systemic level by orthodontic treatment. Both, the ROS formation as well as the AD potential were measured in blood/serum, and the ratio between them was calculated [12] in subjects undergoing orthodontic treatment and in a control group.

The results evidenced a marked short-term systemic increase of ROS as well as an increase in the ratio between ROS and AD, among subjects undergoing orthodontic treatment. In accordance with the study of Olteanu et al. [6] that revealed maximum levels of salivary oxidative stress biomarkers 24 hours after the start of orthodontic treatment, the present study also denoted a significant increase of the systemic (blood/serum) ROS/AD ratio 24 hours after the start of treatment. Similarly to the previous report [6], after 7 days of treatment, a decrease of the ROS/AD ratio to normal values as those measured in the CG was observed also in the present study. A recent study by Buczko et al. [5] evidenced significant changes of the total oxidative status index (ratio between the total oxidative status and total antioxidative status) in unstimulated and stimulated saliva during orthodontic treatment. The authors [5] revealed an increase of the total oxidative status in saliva at 1 week and a significant decrease of it at 24 weeks follow-up, which is in contrast with the results of the present study, as the systemic ROS/AD ratio normalized after 7 days.

It could be hypothesized that oxidative stress during orthodontic treatment might be induced by different factors: local and systemic exposure to heavy metals, inflammation of the periodontal tissues due to poor oral hygiene, and aseptic inflammation in the periodontal ligament due to mechanical force application.

In vitro studies [2, 4] have shown that metal ions such as nickel, cobalt, and chromium, released either from corroded orthodontic brackets and archwires, induce oxidative stress. Despite the smaller corrosion susceptibility of titanium alloys, due to the protective titanium oxide layer, mechanical friction in the contact between bracket and archwire during orthodontic treatment leads to the disruption of the protective titanium oxide layer [18, 19], causing corrosion and release of titanium ions, which might increase ROS production [1]. Likewise, the in vivo study by Buczko et al. [5] explained the increase of ROS/AD ratio in saliva after one week as an effect of heavy metal exposure during orthodontic treatment, since the highest concentration of nickel ions was measured simultaneously.

Also in the present study, patients could have been exposed to nickel, cobalt, chromium, and titanium released from the parts of the orthodontic appliance used, all of which might have induced the systemic elevation of the ROS/AD ratio after 24 hours of orthodontic treatment. However, at the 7 days' time point, contrasting the results of salivary oxidative stress biomarkers [5], the ROS/AD ratio normalized, most probably due to adaptive stress responses and induction of antioxidative endogenous defense. This is in accordance with two other in vivo studies [7, 17] that reported no marked changes of the salivary [7, 17] and gingival crevicular fluid [7] oxidative stress biomarkers after 4-5 weeks and six months of orthodontic treatment. Of note, the contrasting results could also be due to the great variability in the timing of nickel ions increase in saliva, which ranges from 10 minutes to four weeks after orthodontic appliance insertion [20, 21].

A second cause of the significant systemic elevation of ROS and ROS/AD ratio could be the periodontal inflammation induced by increased plaque apposition due to the orthodontic appliance. Although periodontal inflammation has been associated with ROS formation [3], Portelli et al. [17] reported no notable correlation between oxidative stress biomarkers and oral hygiene in patients undergoing orthodontic treatment. Similarly, periodontal inflammation as a cause of oxidative stress could be excluded in the present study, as all the subjects had excellent oral hygiene without any signs of periodontal inflammation at each time point.

A final explanation for the increase in ROS and ROS/AD ratio detected in the present study 24 hours after the start of orthodontic treatment could be a result of the expression of proinflammatory mediators in the periodontal ligament induced by mechanical force application on the tooth. In fact, the mechanism of orthodontic tooth movement with fixed appliances is characterized by a cascade of events, triggered by the strain of the periodontal ligament fibers, leading to an inflammatory process that allows appropriate tissue remodeling. It has been shown that this inflammation might occur only at a subclinical (i.e., molecular level) and might be limited to the alveolar bone, with no systemic consequences in terms of elevation of C-Reactive Protein [22]. However, this does not exclude, that the short-term elevation of systemic ROS and ROS/AD ratio seen in the present study is a consequence of the aseptic inflammation in the periodontal ligament due to force application induced by the orthodontic appliance.

*Limitations of the Study*. It is generally accepted that two or more assays should be utilized to assess oxidative stress status, whenever possible to enhance validity, since each technique measures something different and has its own inherent limitations and no method by itself can be said to be a completely accurate measure of antioxidant status and ROS formation [11]. In the present study ROS and different antioxidants present in the blood as well as their interactions were assessed with FORT and FORD. Although changes in the ROS/AD ratio were observed over time, their main cause(s) could not be determined. In fact, the observed ROS/AD ratio changes can be related to many factors (i.e., endogenous antioxidants activation, inflammation, and blood metal ions), the assessment of which was beyond the scope of the present study. On the other hand, the possible influence of periodontal inflammation on the measured systemic oxidative stress parameters could be excluded, since no signs of inflammation were detected in any of the subjects over the observed period of time, the influence of sterile periodontal inflammation due to force application and blood metal ions content could not be excluded as the cause of increased ROS observed in the TG. In fact, due to ethical reasons it was not feasible to retrieve consecutive larger venous blood samples four times over a period of one week for assessing any possible changes of inflammation mediators as well as heavy metals in venous blood. Moreover, previous studies [23] reported that heavy metal ions (i.e., nickel) are detectable in blood only after long-term exposure.

Given that the results presented here are descriptive and future research is needed for a better understanding of which factors (presence of heavy metals and/or inflammation) have a direct causative impact on increased parameters of ROS and ROS/AD ratio observed in the blood of the treated group. Nevertheless, due to the short-term elevation of oxidative stress parameters during the first week of orthodontic treatment, increased intake of natural antioxidants would be recommended. However, a study on the efficacy of antioxidant treatment during orthodontic therapy should be performed to determine the rational and dosage of their use. In fact, an excess use of antioxidants might also induce harmful health effects [24, 25].

## 5. Conclusions

Orthodontic treatment with fixed appliances might induce systemic oxidative stress, but only in the short-term. In particular, the elevation of ROS and ROS/AD levels is seen only 24 hours after the start of orthodontic treatment, while normalization of the levels occurs within 7 days after archwire insertion most probably due to adaptive endogenous antioxidative response. However, intermittent changes of the ROS and AD levels during orthodontic treatment (i.e., at each archwire reactivation) could not be excluded. Future studies should be performed to confirm the activation of endogenous antioxidant defense (superoxide dismutase, catalase, and glutathione peroxidase activity) as well as the main cause of increased oxidative stress (heavy metal release and/or inflammation) during orthodontic treatment with fixed appliances.

## Figures and Tables

**Table 1:** The FORT and FORD levels among the treated and control groups over the observational period.

Parameter	Group	Baseline	6 hours	24 hours	7 days	Diff.
FORT	TG	1.54 (1.22;2.12)	1.78 (1.22;2.15)	2.05 (1.71;2.26), *a*	1.64 (1.22;2.08), *b*	0.026; *S*
	CG	1.74 (1.40;2.01)	1.71 (1.22;2.01)	1.67 (1.29;1.95)	1.64 (1.29;1.92)	0.100; *NS*
	*Diff.*	0.602; *NS*	0.376; *NS*	0.025; *S*	0.910; *NS*	

FORD	TG	1.19 (0.66;1.50)	1.23 (0.89;1.45)	1.04 (0.51;1.45)	0.96 (0.66;1.49)	0.331; *NS*
	CG	1.19 (0.57;1.42)	1.07 (0.66;1.44)	0.91 (0.66;1.41)	1.20 (0.72;1.57)	0.887; *NS*
	*Diff.*	0.775; *NS*	0.276; *NS*	0.431; *NS*	0.307; *NS*	

Data is presented as median (25th; 75th percentile). *TG*, treated group, n=27; *CG*, control group, n=27. *Diff.*, p-values; significance of the difference between the groups within each time point or over time within either group. Results of the pairwise comparisons between time points: *a*, significantly different from the baseline; *b*, significantly different from 24 hours. *NS*, not statistically significant; *S*, statistically significant.

## Data Availability

The data used to support the findings of this study are available from the corresponding author upon request.
